# The Dental Profession in England

**Published:** 1875-09

**Authors:** W. N. Waite

**Affiliations:** Liverpool, England


### THE DENTAL PROFESSION IN ENGLAND.

BY W. N. WAITE, D. D. S., OF LIVERPOOL, ENGLAND.

*Read before the New York State Dental Society June 1875.*

In order to understand the present state of the dental profession in England, a brief retrospect is necessary; and looking back only about twenty years, we see that a vigorous effort was then made by some eminent men of that period, to establish a "College of Dentists," which should somewhat resemble the existing Colleges in America, and seek ultimately for a goverment charter, to give authority to their diploma: This attempt, after some years of struggling, failed, not because the object was undesirable, nor because any better method of attaining the object was discovered nor on account of any diminution of necessity for some such institution; but, as is too often the case in praiseworthy undertakings, failure arouse from internal dissension and jealousy.

Up to this period, those dentists who desired to stand well before the public as educated practitioners, had resorted to the only available qualification, that had any bearing on their specialty, namely, the certificate of membership of the Royal College of Surgeons.

These men stood well with their medical confreres, and aspired to be leaders among their dental brethren. For some time they worked well in connection with the scheme of the College of Dentists, but when this failed to offer them the distinction they coveted, a new scheme was propounded by which the dental profession was to be tacked on to the tail of the medical profession; derive its qualification from a medical institution, the Royal College of Surgeons, and become to all intents and purposes an appendage rather than a strong, united and independent body.

The Odontological Society which originated mainly in the break up of the College of Dentists, took up this role, and by dint of great labor and undaunted perseverance, at length succeeded, in 1859, in persuading the R. C. S. to institute a dental examination and confer a dental diploma.

The name of the diploma is as follows: "Licentiate of Dental Surgery of the Royal College of Surgeons."

(It may not be known to Americans that, according to British law, and with a view to furnishing funds for the support of the British aristocratic paupers, that no man can sell tea, coffee, tobacco, spirits, or keep a horse, dog, carriage or gun without taking out a license and paying duty. Hence, over every grocery store you may read "Licensed to sell tea, coffee, etc. In like manner, to complete the scheme, and with a view to make the public familiar with the honorable distinction conferred upon certain dentists by the R. C. S., ought they not to be required to paint over their door "Licensed to pull teeth.")

In 1859, the R. C. S. announced that a curriculum of study had been arranged, consisting chiefly of attendance at the usual courses at the medical schools, and that after a certain period of instruction students may present themselves for examination before a board comprised of three surgeons and three dentists.

Further, it was announced that any one whose education commenced prior to the date of this arrangement might, on filling up certain forms of enquiry, be admitted to a modified examination, and thus obtain the diploma. This provision of course covers existing practitioners. In perfect simplicity and good faith, quite a large number of applications were sent in during the four years for which this modified examination was kept open, but the treatment accorded the applicants was both partial and arbitrary. In many cases men of known reputation and unquestioned ability and established practices were refused permission to come up for examination. No reason being assigned for their rejection; while, on the other hand, men were admitted whose connection with dentistry in any shape was only for a few months' duration, and whose fitness for practice was notoriously insufficient. One such case may serve as example: A man who, with his father, had carried on business as a pickle manufacturer, and who had again and again come to grief, so that his character and credit were entirely done for, turned his attention to dentistry. A practitioner who had already obtained the diploma and who now holds the office of dental surgeon to a large hospital, got hold of Mr. Picklemaker and undertook to get him the diploma for the paltry consideration of £10 or about fifty dollars.

By means of some coaching up and a good deal false statement, the plan succeeded, and in a short time Mr. Picklemaker came forth a Licentiate of Dental Surgery of R. C. S. Of course, any thing like professional behavior was out of the question, and, so walking through the streets of the city where he resides, you may see attached to lamp posts and other accessible positions, sign boards announcing his name, profession and qualification in charaters that are unmistakable. This notwithstanding one of the requirements of graduates is that they take an oath not to use any advertisements.

In 1863, this arrangement ceased, and the result of four years nominal admission, upon a mere pretense of examination was, that for all Great Britain 250 were, or probably one-tenth of the profession, made licentiates of the R. C. S.

The real value of the diploma and the appreciation accorded to it by the profession and public, now became evident by the number of students who entered the curriculum appointed as a preparation for the real examination.

The average of ten years from 1863 to 1873, for all Great Britain was seven per annum. Of course, even the most bigoted partisans of this scheme could hardly pretend to be satisfied with this product while the bulk of the profession pointed to it as the natural result of bad management and favoritism. Dissension prevailed extensively, and at length an attempt was made to undo, or rather do over again the work so badly bungled from 1859 to '63. A committee was formed and, after two years wire pulling and wheel turning, a petition was presented to the governing body of the R. C. S., praying that the doors may be reopened to those who were in practice prior to 1859, with a view to their admission to the ranks, now somewhat thinned by death and other causes. It was probably perceived that to answer this request would be to admit past error, and offer a poor solatum to those who had been confessedly mal-treated. So more wire pulling had to be resorted to, and at length a reluctant consent was wrung from the council, during July, 1874.

No sooner said than done! The British Journal, for that month (the acknowledged penny whistle of the Odontological Society) was kept back in order to carry forth with a flourish cf trumpets, the invitation to practitioners.

"Walk up, gentlemen, walk up!"

Now's your time gents, only 10 guineas! The last point very emphatic, for it was freely surmised, and that not without reason, that only the prospect of a shower of gold, would have induced the council to yield the point.

The next month's journal announced that thirty-six applications had been already received from old practitioners, for admission to the examination, which would be held at Christmas last. Of these thirty-six, three were from persons known to the writer, and the kind of reception accorded, was as follows: Two were immediately accepted, on their supplying the proper forms filled up. The third was subjected to a series of impertinent questions, and at last (three weeks after the examination had passed by,) was informed that he would not be admitted.

The case was thus: One of the questions contained in the preliminary form, is, "Do you employ any advertisements?" Now it so happened that all the three had employed advertisements of a very simple and inoffensive kind, within the prescribed period, *but*, the two who were admitted answered no! while the rejected one answered, yes, and thus lost his chance.

When the week of examination came, one of these two was detained at home by domestic trouble. The other having been informed that his examination would be on Friday, Jan. 8, went to London. He found some fifteen youths, waiting with one old practitioner, and after devoting some five hours to answering as many written questions, he was coolly told that his oral examination would take place on that day, week.

Now it should be known that hitherto, written and oral examinations have always been held on the same day or one or two succeeding days, but on this, the first occasion of inviting gentlemen from a distance, they arranged to put a week's interval between, thus necessitating upwards of a week's absence from home, and residence in London, or, two expensive and tedious journeys, to and fro, making six days absence and double cost, where one journey and three days absence would have been ample. The gentleman referred to, declined to go up a second time, but they had fingered his 10 guineas, before announcing the facts to him!

What had become of the other thirty-three?

Such abominable arbitrary and unreasonable behavior, on the part of the examining board, renders the reopening of the examination, nothing more or less than a farce, and so far from serving the interests of the profession, or promoting its advance before the public, it will assuredly make the diploma less popular, and postpone still further, the term for our recognition by Parliament as a body of skillful and competent men. There is this consolation for all who paid to obtain the diploma, viz: that to those already established in practice, it can be of no possible value, inasmuch as the public generally beyond a few here and there, do not so much as know if there be any diploma. The next 10 or 15 years will probably see the number of licentiates so far reduced as to render the parchment valueless to beginners.

It is much to be deplored, that our profession in this country should be hampered and choked and disintegrated through the bungling stupidity of a few earnest men, who through pure selfishness and narrow mindedness persist in holding the reins, notwithstanding their utter inability to drive. Some blame is no doubt due to those who stand by quietly and submit to be ruled by incompetent men, but the question remains what can be done? We have only one center in England, viz: London, and London essays to govern the country in more ways than in dentistry. If London be wrong, there may be suffering and ill consequences to bear, but there is no other rallying point.

It may be asked, why be so anxious for this diploma to be wider spread? only for one reason. If all the really respecta-men held it, and were to unite in a compact strong phalanx we should soon be able to obtain recognition and some amount of protection, such as medicine enjoys, viz: That no one should be entitled to practice dentistry unless duly qualified by the possession of the diploma, but, so long as there are a number of respectable men excluded, from whatever cause, it is only hopeless, to think of obtaining any such recognition.

The absurdity of making it a sine qua non, that no advertisement whatever, should have been used for 15 years past, is more apparent, when one knows, that a considerable sprinkling of the licentiates not only advertise, but do so in the same style, if not to the same extent, as the quacks. True, the Board of Examiners can not prevent the infringement of rule by their alumni, but at least they might make some effort to check the evil, and if that failed, they might strike the name of any advertising licentiate off the list. But nothing is done. So far from taking off the name of even the most notorious advertiser, they not only publish their list, on every possible occassion, but print it in the British Journal, twice over in the same number. Once alphabetically, and once chronologically, to make it appear something worth printing.

The principle adopted by the Board of Examiners in relation to candidates in practice, seems to be, "you must not be known to advertise before you come up, you may do as you like afterwards."

Now it can not be a matter of surprise to any one, that with such disorder and bad management on the part of those who assume the leadership in dental politics, there should be, all sorts of irregularity and confusion among the rank and file.

Hence, while the skilful and competent members of the profession are squabbling and frittering away their best chances of union and recognition, the public are being humbugged and maltreated to a fearful extent by a numerous horde of quacks.

There is scarcely a newspaper in the country that does not contain the announcements of these unscrupulous mountebanks, and they succeed in obtaining a very large share of patronage, thus bringing the practice of dentistry down to the level of a mere trade, in public estimation, and rendering the task of educating the people and elevating the appreciation of high-class operations, all but hopeless. English folks, as a people, have no faith in operative dentistry. When told on every newspaper and railway guide, that they can secure the "very best artificial teeth for 5 shillings each, without extraction of roots or any other painful operation," they naturally conclude to let their teeth do as long as they will and then go in for new ones, since they can be had so cheaply.

Our depot keepers, are partly to blame for this. Their traveling agents, and especially those of our first house, are continually persuading little chemists and others to start dentistry, offering to teach them vulcanite work, and supply them with materials and so on and it is an actual fact, that one of the largest Jewish firms of dental quacks, can obtain and does obtain his materials at our principal depot at a considerable discount, below the price charged to any other practitioner. All this quackery and huckstering would be demolished, or at least, made to hide its head if the well educated and respectable men were strong and united, and determined to obtain a distinct and acknowledged status. But while they remain disunited and seek what recognition they have, at the hands of another, albeit, a kindred society, it is hopeless to look for any improvement. The profession must be degraded; the public must be gulled; the results must be that educated men, will not put their sons into a calling so uncertein and indefinite. And precisely that result obtains at the present time to a large extent.

A gloomy picture, some one will say. So it is. A most unsatisfactory and unpromising state of things. Even the chemists are ahead of us. They are now required to pass an examination before entering on their calling. Whereas, the tinker or chimney sweep, or quack doctor, or anybody who pleases, may hoist a shingle, with dentist, upon it, and stand as good a chance of public favor, as the most skillful and educated man amongst us.

Having thus briefly, detailed the condition of dental politics in England, it is almost superfluous to say that the chief occupation of the English dentist, is making artificial cases.

An apprentice to one of the most respectable practitioners in this city, after five years spent in the workshop, had not even attempted to extract a tooth, much less to fill, or perform any more delicate operation. Should he have time and money to enable him to go through the curriculum for the diploma he will pick up some acquaintance with operatious at the Dental Hospital in London, but if, as is most likely, he will not be able to do this, then, he will commence practice sooner or later on his own account without any knowledge whatever of operative dentistry. Thus it is with nearly all.

Naturally such a one, will cultivate the department which he understands, and so, even among competent and skillful mechanicians, the operative department is almost entirely overlooked. This is evident when one hears a conversation between practitioners. In speaking of their amount of business, you always find, they speak of the numbcr of cases in hand, how many full sets or partial sets, how many teeth they have mounted and the like.

He who tries to teach the value of preserving the natural organs, is constantly met by the remark, "oh! I mean to let my teeth go and have a new set." "Mr. so and so, told me long ago that all my teeth would go and it was no use to try to stop them."

Then further, where attempts are made to overcome this wholesale desolation, they are chiefly confined to amalgam fillings, cheap and nasty, but easy to insert. From this custom, one meets the frequent remark. "Oh! my teeth won t bear gold." Mr. so and so, told me so years ago." Of course if you succeed in overcoming that obstacle, and get alongside the question of cost, you at once seem to be striving after the larger fees and are very likely to be suspected, while Mr. so and so, was implicitly believed.

But let this suffice. There are many very good and able men over here fighting hard for better days, and hope that star in man's brain which never ceases to shine, bids us look forward, and resolve, that for ourselves we will do our utmost, to achieve a better condition, and leave things better than we found them.

